# Job strain as a risk factor for clinical depression: systematic review and meta-analysis with additional individual participant data

**DOI:** 10.1017/S003329171600355X

**Published:** 2017-01-26

**Authors:** I. E. H. Madsen, S. T. Nyberg, L. L. Magnusson Hanson, J. E. Ferrie, K. Ahola, L. Alfredsson, G. D. Batty, J. B. Bjorner, M. Borritz, H. Burr, J.-F. Chastang, R. de Graaf, N. Dragano, M. Hamer, M. Jokela, A. Knutsson, M. Koskenvuo, A. Koskinen, C. Leineweber, I. Niedhammer, M. L. Nielsen, M. Nordin, T. Oksanen, J. H. Pejtersen, J. Pentti, I. Plaisier, P. Salo, A. Singh-Manoux, S. Suominen, M. ten Have, T. Theorell, S. Toppinen-Tanner, J. Vahtera, A. Väänänen, P. J. M. Westerholm, H. Westerlund, E. I. Fransson, K. Heikkilä, M. Virtanen, R. Rugulies, M. Kivimäki, for the IPD-Work Consortium

**Affiliations:** 1 National Research Centre for the Working Environment, DK-2100 Copenhagen Ø, Denmark; 2 Finnish Institute of Occupational Health, FI-00250 Helsinki, Finland; 3 Stress Research Institute, Stockholm University, SE-106 91 Stockholm, Sweden; 4 Department of Epidemiology and Public Health, University College London, London WC1E 6BT, UK; 5 School of Community and Social Medicine, University of Bristol, Bristol BS8 2PS, UK; 6 Institute of Environmental Medicine, Karolinska Institutet, SE-171 77 Stockholm, Sweden; 7 Centre for Occupational and Environmental Medicine, Stockholm County Council, SE-104 22 Stockholm, Sweden; 8 Centre for Cognitive Ageing and Cognitive Epidemiology, University of Edinburgh, Edinburgh EH8 9JZ, UK; 9 Alzheimer Scotland Dementia Research Centre, University of Edinburgh, Edinburgh EH8 9JZ, UK; 10 Department of Occupational and Environmental Medicine, Bispebjerg University Hospital, DK-2400 Copenhagen, Denmark; 11 Federal Institute for Occupational Safety and Health (BAuA), D-10317 Berlin, Germany; 12 INSERM, U1085, Research Institute for Environmental and Occupational Health (IRSET), Epidemiology in Occupational Health and Ergonomics (ESTER) Team, F-49000, Angers, France; 13 University of Angers, Epidemiology in Occupational Health and Ergonomics (ESTER) Team, F-49000, Angers, France; 14 Netherlands Institute of Mental Health and Addiction, 3521 VS Utrecht, The Netherlands; 15 Department of Medical Sociology, University of Düsseldorf, 40225 Düsseldorf, Germany; 16 National Centre for Sport & Exercise Medicine, Loughborough University, Loughborough LE11 3TU, UK; 17 Institute of Behavioral Sciences, University of Helsinki, FI-00014 Helsinki, Finland; 18 Department of Health Sciences, Mid Sweden University, SE-851 70 Sundsvall, Sweden; 19 Department of Public Health, University of Helsinki, FI-00014 Helsinki, Finland; 20 Unit of Social Medicine, Frederiksberg University Hospital, DK-2000 Copenhagen, Denmark; 21 Department of Psychology, Umeå University, SE-901 87 Umeå, Sweden; 22 The Danish National Centre for Social Research, DK-1052 Copenhagen, Denmark; 23 The Netherlands Institute for Social Research, 2515 XP The Hague, The Netherlands; 24 Department of Psychology, University of Turku, FI-20014 Turku, Finland; 25 Inserm U1018, Centre for Research in Epidemiology and Population Health, F-94807 Villejuif, France; 26 Folkhälsan Research Center, FI-00290 Helsinki, Finland; 27 Nordic School of Public Health,SE-402 42Göteborg, Sweden; 28 Department of Public Health, University of Turku, FI-20014 Turku, Finland; 29 Turku University Hospital, FI-20520 Turku, Finland; 30 Occupational and Environmental Medicine, Uppsala University, SE-751 85 Uppsala, Sweden; 31 School of Health and Welfare, Jönköping University, SE-551 11 Jönköping, Sweden; 32 Department of Health Services Research and Policy, London School of Hygiene and Tropical Medicine, London WC1H 9SH, UK; 33 Clinical Effectiveness Unit, The Royal College of Surgeons of England, London WC2A 3PE, UK; 34 Department of Public Health and Department of Psychology, University of Copenhagen, DK-1353 Copenhagen, Denmark; 35 Clinicum, Faculty of Medicine, University of Helsinki, FI-00014 Helsinki,Finland

**Keywords:** Observational studies, occupational health, work stress

## Abstract

**Background:**

Adverse psychosocial working environments characterized by job strain (the combination of high demands and low control at work) are associated with an increased risk of depressive symptoms among employees, but evidence on clinically diagnosed depression is scarce. We examined job strain as a risk factor for clinical depression.

**Method:**

We identified published cohort studies from a systematic literature search in PubMed and PsycNET and obtained 14 cohort studies with unpublished individual-level data from the Individual-Participant-Data Meta-analysis in Working Populations (IPD-Work) Consortium. Summary estimates of the association were obtained using random-effects models. Individual-level data analyses were based on a pre-published study protocol.

**Results:**

We included six published studies with a total of 27 461 individuals and 914 incident cases of clinical depression. From unpublished datasets we included 120 221 individuals and 982 first episodes of hospital-treated clinical depression. Job strain was associated with an increased risk of clinical depression in both published [relative risk (RR) = 1.77, 95% confidence interval (CI) 1.47–2.13] and unpublished datasets (RR = 1.27, 95% CI 1.04–1.55). Further individual participant analyses showed a similar association across sociodemographic subgroups and after excluding individuals with baseline somatic disease. The association was unchanged when excluding individuals with baseline depressive symptoms (RR = 1.25, 95% CI 0.94–1.65), but attenuated on adjustment for a continuous depressive symptoms score (RR = 1.03, 95% CI 0.81–1.32).

**Conclusions:**

Job strain may precipitate clinical depression among employees. Future intervention studies should test whether job strain is a modifiable risk factor for depression.

## Introduction

Depression is a leading cause of disability associated with considerable costs in terms of lost quality of life and productivity (Alonso *et al.* 2004*c*; Whiteford *et al.* 2013). The 12-month prevalence of depression in Europe is estimated at 7% (Wittchen *et al.* 2011) and studies suggest that up to 41% will suffer from depression at some point during their life (Moffitt *et al.* 2010). The aetiology of depression is multifactorial, involving an interplay of biological, environmental and psychological factors such as genetics, socio-economic disadvantage and severe adverse life events (Kendler *et al*. 2002, 2006). Whether psychosocial factors in the work environment contribute to the development of depression is unclear although an increasing number of prospective studies suggest that this might be the case (Bonde, 2008; Netterstrøm *et al.* 2008; Siegrist, 2008; Theorell *et al.* 2015). The majority of these studies have examined job strain, a work stressor characterized by the combination of high job demands and low job control. According to at least four systematic reviews job strain is associated with an increased risk of depression (Bonde, 2008; Netterstrøm *et al.* 2008; Siegrist, 2008; Theorell *et al.* 2015).

However, the clinical relevance of these findings remains uncertain for several reasons. First, in most studies of job strain and depression, investigators have measured the outcome using self-rated symptom scales with no corroborating evidence from clinical diagnoses (Bonde, 2008; Netterstrøm *et al.* 2008; Siegrist, 2008; Theorell *et al.* 2015). Second, potential publication bias amplified by the availability of several alternative ways to define job strain (Landsbergis *et al.* 2000; Netterstrøm *et al.* 2008; Kivimäki *et al.* 2013) may have led to an overestimation of the effect of job strain. Third, there is a lack of sufficiently powered studies to determine consistency of the association between job strain and depression in subgroups, in particular across socio-economic status (SES) groups.

To address these shortcomings, we present the results of a systematic review and meta-analysis of the published literature in combination with unpublished data from studies participating in the Individual-Participant-Data Meta-analysis in Working Populations (IPD-Work) consortium (Kivimäki *et al.* 2012). In doing so, we provide the first large-scale study of the association between job strain and clinically diagnosed depression. To minimize selective reporting and other *post-hoc* decision-making biases, we published a detailed protocol for the individual participant data (IPD) analysis, in which we listed the studies to be included, defined job strain and depression, and presented a detailed analytical plan prior to commencement of the data analysis (Madsen *et al.* 2014).

## Method

### Published studies

#### Search strategy and selection criteria

In accordance with the Preferred Reporting Items for Systematic Reviews and Meta-Analyses (PRISMA) guidelines (Moher *et al.* 2009), we conducted a systematic search of the literature limited to research on humans in PubMed and PsycNET (to September 2015). We used the following search terms: [‘job strain’ OR (‘demands’ AND ‘control’)] AND (‘depression’ OR ‘depressive disorder’). We also scrutinized the reference lists of all relevant publications identified and those of key publications. In addition, using the Institute of Scientific Information Web of Science we searched references citing the retrieved articles (to October 2015).

Two authors (I.E.H.M., R.R.) independently reviewed titles and abstracts to retrieve potentially relevant studies. Selected full articles were scrutinized, and included if they met the following criteria: published in English; original contribution of empirical study published in a peer-reviewed journal; prospective design; examined the effect of job strain measured at the individual level (no ecological studies); used clinically diagnosed depression, assessed by diagnostic interview or hospital records, as the outcome. A diagnostic interview is regarded the ‘gold standard’ for assessing clinical depression (Drill *et al.* 2015) and hospital records provide diagnostic codes of the disorders. We did not include antidepressant treatment as these medications are used to treat conditions other than depression, for example anxiety disorders and neuropathic pain (Gardarsdottir *et al.* 2007). We also excluded measures related to labour market attachment, such as sickness absence or disability pensioning due to depression, as they are not only defined by impairment, but depend also on non-medical factors, such as disability pension regulations, the work environment and workplace willingness to accommodate the disability.

#### Data extraction and quality assessment

From each eligible article we extracted the following: name of the first author, year of baseline and follow-up, study location (country), number of participants, number of depression cases, mean age of participants, proportion of women, method of depression ascertainment, covariates included in the adjusted models, and estimate of relative risk, odds ratios or hazard ratios (HRs) with 95% confidence intervals (CIs) for the association between job strain *v*. no job strain and depression. If the comparison for job strain *v*. no job strain was not reported, we contacted principal investigators to obtain this risk estimate. The quality of each included study was assessed by I.E.H.M. and R.R. using the Newcastle–Ottawa scale (Wells *et al.* 2000). Any differences were resolved through discussion.

#### Statistical analyses

We combined study-specific risk estimates for the association between job strain and clinical depression in each study using meta-analytic techniques. If more than one statistical model was published, we included the risk estimate from a sociodemographic-adjusted model (adjusting for example, sex, age, marital status, education) to increase comparability with the IPD. We pooled the study-specific effect estimates and their standard errors in random-effects meta-analysis and assessed heterogeneity with the *I*^2^ statistic and Cochran's Q test (tau-squared). We used the R package meta (Schwarzer, 2012) to perform the meta-analyses. All statistical tests used a significance level of *p* < 0.05.

### Unpublished IPD

#### Study inclusion

We included unpublished IPD from 14 IPD-Work cohort studies conducted in Denmark, Sweden, Finland and the UK: The Copenhagen Psychosocial Questionnaire (COPSOQ) studies I and II, the Danish Work Environment Cohort Studies (DWECS) from 2000 and 2005, the Finnish Public Sector Study (FPS), the Health and Social Support Study (HeSSup), the Intervention Project on Absence and Well-being (IPAW) study, the Burnout, Motivation and Job Satisfaction (PUMA) study, the Swedish Longitudinal Occupational Survey of Health (SLOSH) from 2006 and 2008, the Still Working study, the Whitehall II study, and the Work, Lipids, Fibrinogen studies from Norrland (WOLF-N) and Stockholm (WOLF-S). The studies were selected from the cohorts participating in the IPD-Work consortium because they included data on job strain and hospital records of treatment for depression.

Within each study, we used the first wave of data collection where job strain was measured and participants were eligible for inclusion if they were gainfully employed at baseline. We excluded participants with missing data on sex, age, cohabitation, SES or hospital treatment, and those hospitalized for depression before study baseline. All studies were approved by the relevant local or national ethics committees and all participants gave informed consent to participate. A description of the study and participant selection is given in online Supplementary Appendices S1 and S2.

#### Measurement of job strain

Job strain was measured with questions from the validated job-content and demand–control questionnaires (Fransson *et al.* 2012*a*). A detailed description of the job-strain measure, including its validation and harmonization across the IPD-Work studies, has been published (Fransson *et al.* 2012*a*). Briefly, participants were questioned about the demands of their job (e.g. excessive amounts of work, conflicting demands, or insufficient time) and their level of control (e.g. decision freedom or learning new things at work). For each participant, we calculated mean response scores for job-demand items and job-control items. The Pearson correlation coefficient between the applied harmonized scales and the complete versions was greater than *r* = 0.9, except for one study in which *r* = 0.8.

Having dichotomized demands and control into high and low by their study-specific medians, we defined job strain as the combination of high demands and low control. We analysed data comparing participants with job strain with those without job strain (all other combinations of demands and control). This approach is consistent with the original theoretical model of job strain (Karasek & Theorell, 1990), although several alternative ways of analysing job strain data exist (Landsbergis *et al.* 2000). In planned sensitivity analyses (Madsen *et al.* 2014) we included two such alternative approaches: the quadrant method, that is comparing the job strain group and the groups with active (high demands and high control) and passive jobs (low demands and low control) with participants with low demands and high control (low strain); and using demands and control as separate continuous variables.

#### Ascertainment of depression

Depression was ascertained from hospital registers for in- and out-patient treatment (online Supplementary Appendix S3). We included all hospital contacts with a principal diagnosis of depression based on the International Classification of Diseases (ICD). As described in the protocol and online Supplementary Table S1, incident cases were primarily defined using ICD-10, codes F32 and F33 (Madsen *et al.* 2014).

#### Covariates

We included age, sex, cohabitation and SES as potential confounders because they are important risk factors for depression (Alonso *et al.* 2004*b*) and may also be related to job strain. SES was measured by occupation, except in HeSSup, where we used education, and categorized as low (routine and manual occupations or basic education), intermediate (non-manual intermediate occupations or vocational education) or high (higher managerial, administrative and professional occupations or university-level education).

We also included self-reported depressive symptoms at baseline (online Supplementary Appendix S4). Self-reported depressive symptoms could act either as a confounder (by affecting the self-reported data on job strain and being a risk factor for clinical depression) or a mediator (by being part of the causal pathway between job strain and hospital-treated depression). In accordance with the study protocol (Madsen *et al.* 2014), we accounted for baseline depressive symptoms in two different ways: by adjusting for depressive symptoms (continuous score); and by excluding participants with depressive symptoms (defined as the top 20% of the depressive symptom score in each study). We also measured self-reported somatic disease (coronary heart disease, stroke, cancer, chronic obstructive pulmonary disease, musculoskeletal disorders or diabetes) at baseline. Somatic disease may also be conceptualized as a confounder (somatic disease increasing job strain levels) or mediator (job strain is a risk factor for cardiometabolic and musculoskeletal disorders) (Hauke *et al.* 2011; Steptoe & Kivimäki, 2013; Nyberg *et al.* 2014). In eight cohort studies, repeat measurements of job strain and depressive symptoms were available allowing us additionally to examine temporal associations between the two.

#### Statistical analyses

We combined study-specific risk estimates for the association between job strain and clinical depression in each study using meta-analysis. We modelled job strain as a binary exposure (job strain *v*. no job strain) and analysed associations with the first episode of hospital-treated depression after baseline for each study using Cox proportional hazards regression. Participants were followed from job strain assessment to the first episode of hospital-treated depression, death, migration (available in Danish data only) or end of follow-up, whichever came first. There were no systematic differences in the study-specific risk estimates by length of follow-up, indicating that the proportional hazards assumption was met.

Minimally adjusted HRs and 95% CIs for the association between job strain and hospital-treated depression were adjusted for age, sex and cohabitation (main model). We further adjusted the association for SES and baseline depressive symptoms score to examine if they explained the association. These factors were not included in the main models to avoid potential over-adjustment because SES is conceptually intertwined with job strain (Johnson & Hall, 1995) and depressive symptoms could be part of a causal pathway between job strain and clinical depression.

We also examined if the risk estimate was similar when excluding participants with depressive symptoms or somatic disease at baseline and if the association differed between men and women, across age groups (⩽35, 36–49, 50+ years) or SES (low *v*. intermediate/high). Following Strengthening the Reporting of Observational Studies in Epidemiology (STROBE) guidelines (Vandenbroucke *et al.* 2007), effect modification was examined both as departure from additivity and departure from multiplicativity.

In additional (*post-hoc*) analyses we explored whether the association with repeat exposure to job strain was stronger than that seen for a single exposure measurement; whether associations between job strain and depressive symptoms were bi-directional (using both negative binomial, and meta-analytic structural equation modelling); whether there was a statistically significant interaction (departure from multiplicativity) between demands and control in their association with hospital-treated depression. Using meta-regression we further explored if there were systematic differences according to year of study baseline or study country of origin. The number of included studies varied in sensitivity analyses due to lack of depression cases in some subgroups or data unavailability.

We pooled study-specific effect estimates and their standard errors in random-effects meta-analysis and assessed heterogeneity with the *I*^2^ statistic and Cochran's Q test (tau-squared). We used SAS (version 9.3; USA) to analyse the study-specific datasets and R packages meta (Schwarzer, 2012), metafor (Viechtbauer, 2010) and metaSEM (Cheung, 2015) to perform meta-analyses, meta-regression and meta-analytic structural equation modelling, respectively. All statistical tests used a significance level of *p* < 0.05.

### Ethics

This work was conducted in accordance with the Declaration of Helsinki. All studies were approved by the relevant local or national ethics committees and all participants gave informed consent to participate.

## Results

### Published studies

#### Selection of studies and participants in published studies

In the systematic review we identified 1135 potentially eligible records. We assessed the eligibility of 32 full-text articles and found six eligible studies (Table 1, flowchart in online Supplementary Appendix S5). Three studies provided risk estimates for job strain defined dichotomously, and for the other three studies [Psychological risk factors in the work environment and biological mechanism for the development of stress, burnout and depression (PRISME), Netherlands Mental Health Survey and Incidence Study (NEMESIS) and Santé et Itinéraire Professionnel (SIP)] (Plaisier *et al.* 2007; Grynderup *et al.* 2012; Niedhammer *et al.* 2015) we obtained such risk estimates from principal investigators.

**Table 1.** Characteristics of included published studies on job strain and clinical depression

Reference	Country, population	Total *n* (cases)	Year baseline–follow-up	Exposure	Outcome	Results, OR (95% CI)	Covariates in model	Age, years	% Women	% Follow-up
Grynderup *et al.* (2012)	Denmark, PRISME (public sector employees)	3110 (58)	2007–2009	Median cut-off	SCAN interview, ICD-10-DCR, trained lay interviewers	2.52 (1.49–4.27)^a^	Sex, age, marital status	Mean = 45.1, s.d. = 10.2	78.3	71.8
Niedhammer *et al.* (2015)	France, SIP (representative)	4855 (198)	2006–2010	Median cut-off	MINI, DSM-IV	1.71 (1.22–2.42)^a^	Sex, age, marital status	Mean = 39.9, s.d. = 9.7	44.4	81.0
Plaisier *et al.* (2007)	Netherlands, NEMESIS (representative)	2610 (117)	1997–1999	Median cut-off	CIDI, DSM-III-R	1.70 (1.14–2.52)^a^	Sex, age, marital status	Mean = 39.6, s.d. = 9.8	42.2	87.0
Shields (2006)	Canada, National Population Health Survey (representative)	6125 (143) men; 5886 (262) women	1994/1995–2002 (2 years of follow-up in each cycle)	Job strain ratio (demands/decision latitude) of 1.2 or higher; 0.8–1.2 = medium strain; 0.8 or lower = low strain	CIDI	Men: high *v*. low, 2.4 (1.7–3.5) Women: high *v*. low, 1.5 (1.2–2.0)	Occupation, working hours, shift work, self-employment, age, marital status, presence of children in the household, personal income, education, heavy monthly drinking and low emotional support	Not reported	50.9	90.3
Virtanen *et al.* (2012)	UK, the Whitehall II Study (civil servants)	2123 (66)	1991–1999	Quadrant model	CIDI, adapted for self-administered computerized interview (UM-CIDI)	1.04 (0.46–2.39)	Adjusted for age and sex	Mean = 46.7 years, s.d. = 4.8	30.6	85.8
Wang *et al.* (2012)	Canada, randomly selected employees in Alberta	2752 (70)	2008–2001	Job strain ratio above 75th percentile	CIDI-Auto by trained lay-interviewers	1.33 (0.65–2.75)	Education, income, supervisor support, co-worker support, working hours, effort–reward imbalance, job insecurity, family-to-work conflict	Mean = 42.6, s.d. = 0.21	43.8	77.0

OR, Odds ratio; CI, confidence interval; PRISME, Psychological risk factors in the work environment and biological mechanism for the development of stress, burnout and depression; SCAN, Schedules for Clinical Assessment in Neuropsychiatry; ICD-10-DCR, International Classification of Diseases 10th revision: diagnostic criteria for research; s.d., standard deviation; SIP, Santé et Itinéraire Professionnel; MINI, Mini International Neuropsychiatric Interview; DSM-IV, Diagnostic and Statistical Manual of Mental Disorders, 4th edition; NEMESIS, Netherlands Mental Health Survey and Incidence Study; CIDI, Composite International Diagnostic Interview; DSM-III-R, Diagnostic and Statistical Manual of Mental Disorders, 3rd edition revised.

a Estimate for job strain obtained from authors.

#### Job strain and clinical depression in published studies

**Fig. 1.**
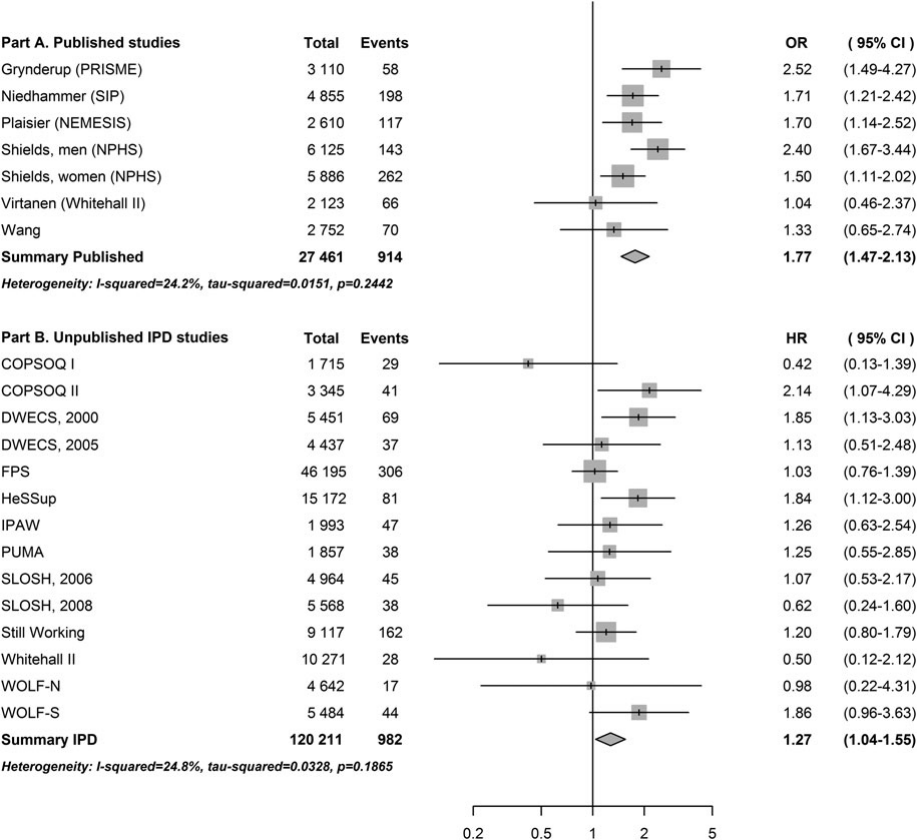
Association between job strain and clinical depression in published (*A*) and unpublished (*B*) data. OR, Odds ratio; CI, confidence interval; PRISME, psychological risk factors in the work environment and biological mechanism for the development of stress, burnout and depression; SIP, Santé et Itinéraire Professionnel; NEMESIS, Netherlands Mental Health Survey and Incidence Study; NPHS, National Population Health Survey; IPD, individual participant data; HR, hazard ratio; COPSOQ, Copenhagen Psychosocial Questionnaire; DWECS, Danish Work Environment Cohort Study; FPS, Finnish Public Sector Study; HeSSup, Health and Social Support Study; IPAW, Intervention Project on Absence and Well-being; PUMA, Burnout, Motivation and Job Satisfaction Study; SLOSH, Swedish Longitudinal Occupational Survey of Health; WOLF-N, Work, Lipids, Fibrinogen Study from Norrland; WOLF-S, Work, Lipids, Fibrinogen Study from Stockholm. ORs for PRISME, SIP and NEMESIS obtained through principal investigators. HRs in IPD-Work studies are adjusted for age, sex and cohabitation at baseline.

We identified 914 cases of clinically diagnosed depression in 27 461 participants (incidence 332.8 per 10 000 participants) from the published studies. Job strain was associated with an increased risk of clinical depression (odds ratio = 1.77, 95% CI 1.47–2.13, Fig. 1*A*). The association in published studies was virtually identical, when including only studies of good quality (odds ratio = 1.78, 95% CI 1.46–2.17, see online Supplementary Appendix S6 for quality assessment).

### Unpublished individual participant studies

#### Selection of studies and participants in IPD

From the unpublished IPD we excluded 710 individuals (0.6%) with hospital-treated depression before baseline. After further excluding 4592 participants (3.7%) with missing data on job strain, age, sex, cohabitation, SES or hospital treatment, the population comprised 120 211 individuals. The baseline mean age was 43.4 years, and 58.5% of participants were women. The prevalence of job strain was 16.6% (Table 2).

**Table 2.** Characteristics of the study population for the unpublished studies

Study	Country	Baseline year	Person-years	Mean length of follow-up, years (s.d.)	Number with incident hospital-treated depression	Incidence rate, cases per 10 000 person years	Number with job strain (%)	Mean age at baseline, years (s.d.)	Number of women (%)	Number cohabiting (%)
COPSOQ I	Denmark	1997	24 760.8	14.4 (2.4)	29	11.7	352 (20.5)	40.8 (10.6)	828 (48.3)	1369 (79.8)
COPSOQ II	Denmark	2004–2005	26 222.2	7.8 (1.0)	41	15.6	474 (14.2)	42.8 (10.2)	1741 (52.0)	2639 (78.9)
DWECS 2000	Denmark	2000	63 301.6	11.6 (1.9)	69	10.9	1215 (22.3)	41.8 (11.0)	2543 (46.7)	4323 (79.3)
DWECS 2005	Denmark	2005	30 886.5	7.0 (0.8)	37	12.0	827 (18.6)	43.1 (10.6)	2240 (50.5)	3549 (80.0)
FPS	Finland	2000	445 421.4	9.6 (1.0)	306	6.9	7488 (16.2)	44.5 (9.4)	37 400 (81.0)	35 043 (75.9)
HeSSup	Finland	1998	105 411.4	6.9 (0.5)	81	7.7	2615 (17.2)	39.8 (10.2)	8388 (55.3)	12 074 (79.6)
IPAW	Denmark	1996–97	30 565.1	15.3 (2.9)	47	15.4	350 (17.6)	41.2 (10.5)	1332 (66.8)	1490 (74.8)
PUMA	Denmark	1999	23 709.6	12.8 (1.8)	38	16.0	283 (15.2)	42.7 (10.2)	1535 (82.7)	1466 (78.9)
SLOSH 2006	Sweden	2006	28 271.7	5.7 (0.4)	45	15.9	984 (19.8)	47.4 (10.8)	2 647(53.3)	3855 (77.7)
SLOSH 2008	Sweden	2008	19 820.5	3.6 (0.3)	38	19.2	1060 (19.0)	47.8 (10.7)	3033 (54.5)	4405 (79.1)
Still Working	Finland	1986	195 807.9	21.5 (3.9)	162	8.3	1416 (15.5)	40.9 (9.1)	2067 (22.7)	6441 (70.6)
Whitehall II	UK	1985–1988	251 222.9	24.5 (3.8)	28	1.1	1441 (14.0)	44.4 (6.1)	3397 (33.0)	7622 (74.1)
WOLF-N	Sweden	1996–98	53 834.5	11.6 (1.1)	17	3.2	595 (12.8)	44.0 (10.3)	777 (16.7)	3624 (78.1)
WOLF-S	Sweden	1992–95	79 170.9	14.4 (2.0)	44	5.6	906 (16.5)	41.7 (11.0)	2378 (43.4)	3978 (72.5)
Total		1985–2008	1 378 406.8	14.3 (2.0)	982	7.1	20 006 (16.6)	43.4 (9.6)	70 306 (58.5)	91 878 (76.4)

s.d., Standard deviation; COPSOQ, Copenhagen Psychosocial Questionnaire; DWECS, Danish Work Environment Cohort Study; FPS, Finnish Public Sector Study; HeSSup, Health and Social Support Study; IPAW, Intervention Project on Absence and Well-being; PUMA, Burnout, Motivation and Job Satisfaction Study; SLOSH, Swedish Longitudinal Occupational Survey of Health; WOLF-N, Work, Lipids, Fibrinogen Study from Norrland; WOLF-S, Work, Lipids, Fibrinogen Study from Stockholm.

#### Job strain and clinical depression in IPD

We identified 982 first episodes of hospital-treated depression over 1 378 407 person years of follow-up (mean 14.3 years, s.d. 2.0 years; incidence 7.1 per 10 000 person-years). There were 196 cases in the 20 008 participants with job strain and 786 in the 100 203 participants without job strain (relative risk = 1.25). After adjustment for sociodemographic factors, job strain was associated with an increased risk of clinical depression (HR = 1.27, 95% CI 1.04–1.55, Fig. 1*B*).

#### Pre-planned sensitivity analyses

**Fig. 2.**
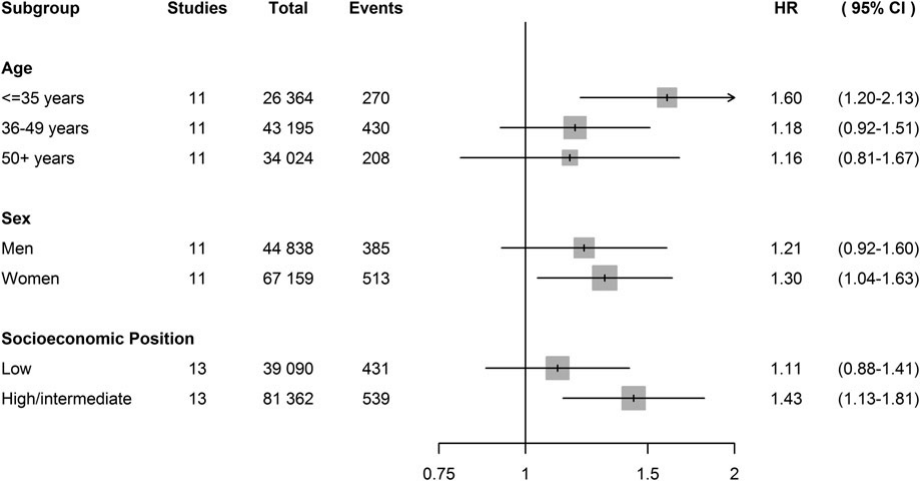
Association between job strain and clinical depression in subgroups. Hazard ratios (HRs) are adjusted for age, sex and cohabitation at baseline where relevant. CI, Confidence interval; COPSOQ, Copenhagen Psychosocial Questionnaire; WOLF-N, Work, Lipids, Fibrinogen Study from Norrland; PUMA, Burnout, Motivation and Job Satisfaction. Studies containing subgroups without depression cases were not included in the subgroup analysis – for age: COPSOQ I, Whitehall II, WOLF-N; for sex: COPSOQ I, PUMA, WOLF-N; for socio-economic status: COPSOQ I.

**Fig. 3.**
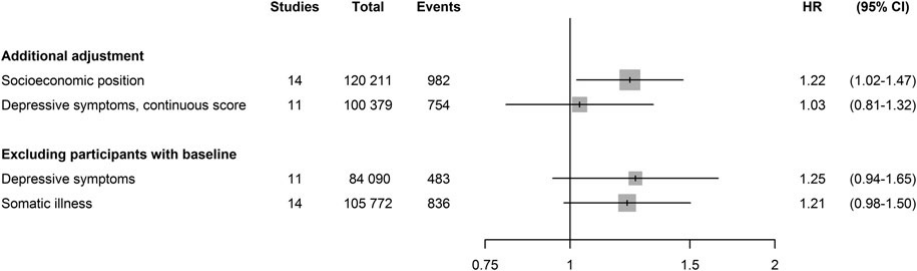
Association between job strain and clinical depression after additional adjustments and exclusions. Hazard ratios (HRs) are adjusted for age, sex and cohabitation at baseline. CI, Confidence interval; WOLF-N, Work, Lipids, Fibrinogen Study from Norrland; WOLF-S, Work, Lipids, Fibrinogen Study from Stockholm. Data on depressive symptoms were not available for Still Working, WOLF-N and WOLF-S.

Fig. 2 and Table 3 show that analyses stratified by age, sex and SES in the IPD studies yielded similar estimates across subgroups. Fig. 3 shows that adjustment for SES, in addition to age, sex and cohabitation, did not substantially change the association between job strain and depression (HR = 1.22, 95% CI 1.02–1.47). After excluding participants with depressive symptoms or with somatic illness at baseline, the association also remained similar. However, after adjustment for baseline depressive symptoms (as a continuous score) the association disappeared (HR = 1.03, 95% CI 0.81–1.32).

**Table 3.** Effect modification of the association between job strain and hospital-treated depression by age, sex and socio-economic status^a^

	Hazard ratio (95% confidence interval)			*p*	
	No job strain	Job strain	Job strain *v*. no job strain in each subgroup	Departure from additivity	Departure from multiplicativity
Age, years				0.8761	0.7418
18–35	1.00 (reference)	1.66 (1.25–2.21)	1.60 (1.20–2.13)		
35–49	1.08 (0.89–1.31)	1.21 (0.85–1.72)	1.18 (0.92–1.51)		
50+	0.74 (0.60–0.92)	0.75 (0.56–1.01)	1.16 (0.81–1.67)		
Sex					
Men	1.00 (reference)	1.20 (0.92–1.58)	1.21 (0.92–1.60)		
Women	1.00 (0.79–1.27)	1.43 (0.99–2.07)	1.30 (1.04–1.63)		
Socio-economic status				0.3187	0.1381
Intermediate/high	1.00 (reference)	1.45 (1.14–1.85)	1.43 (1.13–1.81)		
Low	1.42 (1.16–1.72)	1.58 (1.21–2.05)	1.11(0.88–1.41)		

COPSOQ, Copenhagen Psychosocial Questionnaire; WOLF-N, Work, Lipids, Fibrinogen Study from Norrland; PUMA, Burnout, Motivation and Job Satisfaction.

a Studies containing subgroups without depression cases were not included in the subgroup analysis – for age: COPSOQ I, Whitehall II, WOLF-N; for sex: COPSOQ I, PUMA, WOLF-N; for socio-economic status: COPSOQ I.

As specified in the study protocol (Madsen *et al.* 2014), we examined two alternative operationalizations of job strain (online Supplementary Appendix S7). Using the four quadrants of job strain, the risk of depression was increased for participants with job strain and those with passive work (low demands, low control) compared with low strain. When entering demands and control as continuous variables we found that low control was associated with increased risk of depression but high work demands were not. There was no statistical interaction between demands and control.

#### Supplemental analyses

To examine the association between persistent job strain and hospital-treated depression, we used a subsample of studies with two measurements of job strain, on average 4.8 years apart, and started follow-up for depression after the second measurement. The results supported a dose–response relationship (*p* = 0.03), with the highest depression risk in participants reporting job strain at both measurements (HR = 1.56, 95% CI 0.99–2.45) and more modest among those reporting exposure to job strain only once (HR = 1.23, 95% CI 0.88–1.71) (online Supplementary Appendix S8). We found no indication of effect modification of the association between job strain and hospital-treated depression by year of study baseline or country of origin (*p* = 0.99 and 0.57, respectively).

To clarify the temporal order of the association between job strain and depressive symptoms we examined their bi-directional associations. In participants free of depressive symptoms at baseline, job strain predicted depressive symptoms at follow-up. The age-, sex- and cohabitation-adjusted relative risk for job strain *v*. no job strain was 1.39 (95% CI 1.23–1.57), an association which remained after adjustment for baseline depressive symptoms (continuous score: 1.16, 95% CI 1.07–1.25). Participants with depressive symptoms but no job strain at baseline were, however, also more likely to report job strain at follow-up with a relative risk of 1.46 (95% CI 1.36–1.57) (online Supplementary Appendix S9). These bi-directional associations were supported by the meta-analytic structural equation modelling (online Supplementary Appendix S9, Supplementary Fig. S2).

## Discussion

In this systematic review and meta-analysis of published and unpublished data, job strain was associated with an increased risk of clinically diagnosed depression. The relative risk was 1.77-fold in published studies with diagnostic interviews as the outcome and 1.27-fold for our harmonized IPD based on first episodes of hospital-treated clinical depression. The association between job strain and hospital-treated depression did not differ by sex, age or SES and remained largely unchanged in a series of sensitivity analyses, except after adjustment for continuous depressive symptoms score.

Our findings accord with previous reviews of the published literature that showed an association between job strain and depression measured much more heterogeneously, primarily using self-rated symptom scales (Bonde, 2008; Netterstrøm *et al.* 2008; Siegrist, 2008; Theorell *et al.* 2015). The most recent review, including studies until June 2013, reported an odds ratio of 1.74 for a composite outcome of depressive symptoms and depressive disorders (95% CI 1.53–1.96), virtually identical to our estimate for clinically diagnosed depression in the published data.

The reasons for the stronger association between job strain and clinical depression in the published studies compared with unpublished IPD may relate to at least two factors. First, the definition of the outcome in IPD studies was hospital-treated depression. Because many depressive episodes are not treated (Wittchen & Jacobi, 2005) or treated exclusively in primary care (Alonso *et al.* 2004*a*), the cases included here may differ from other general population cases of clinical depression. Research suggests that clinical decision making regarding depression treatment depends on patient factors such as symptom severity, substance use and social functioning and social relations (Hutschemaekers *et al.* 2014). Also the availability of psychiatric care beds, which varies substantially between countries (OECD, 2016), could affect whether patients get hospitalized. The published studies, in contrast, included also untreated (and primary care-treated), episodes of depression. This may partially explain the stronger association with job strain, if the effects of job strain are more pronounced in relation to milder, less complicated cases of depression. Second, it is possible that the estimate from the published studies was inflated by publication bias. Indeed, previous analyses of the IPD-Work consortium (including similar individual participant datasets as in the present analysis) suggested publication bias in relation to job strain and incident coronary heart disease; the HR being 1.43 (95% CI 1.15–1.77) in those IPD-Work studies that had previously published this finding but 1.16 (95% CI 1.02–1.32) in IPD-Work studies which had not published such analyses (Kivimäki *et al.* 2012; Steptoe & Kivimäki, 2012).

Our findings support previous studies suggesting that effects of job strain may accumulate (Wang *et al.* 2009; Stansfeld *et al.* 2012) and that chronic exposure to job strain may be related to greater risks than exposure at a single point in time. This was also observed in our supplementary analysis, where we found that the risk of hospital-treated depression increased with each report of job strain in a dose–response manner.

Our sensitivity analysis showed that not only job strain but also passive jobs (low demands and low control) were associated with increased depression risk. In earlier work of the demand–control model it has been speculated that passive jobs may be related to experiences of helplessness (Karasek & Theorell, 1990), a psychological phenomenon contributing to the risk of depression (Seligman, 1975). Our findings are consistent with this suggestion, but caution is needed in interpreting these results as they emerged from explorative and not hypothesis-testing analyses.

The association of job strain and risk of depression may be different for different job or social groups. To examine this possibility, we tested effect modification by SES, but found no statistical evidence to support this. Further research is needed for more detailed analyses on effect modification by job and social groups and other factors.

When adjusting for the continuous depressive symptoms score in the individual participant datasets, the association between job strain and hospital-treated depression disappeared. The interpretation of this result is not straightforward because depressive symptoms could mediate or confound the association. A temporal sequence from job strain to depressive symptoms to hospital-treated depression, consistent with mediation, is supported by previous studies showing that job strain predicted depressive symptoms (Bonde, 2008; Netterstrøm *et al.* 2008; Siegrist, 2008; Theorell *et al.* 2015), and by our supplementary analysis showing that job strain predicts the onset of depressive symptoms at follow-up. These findings support the status of job strain as a factor potentially increasing the risk for depressive disorder. However, we also found that among participants with no job strain at baseline, depressive symptoms predicted the onset of job strain at follow-up, suggesting that depressed individuals may be more prone to experience job strain than their non-depressed counterparts. Consequently, as also supported by our meta-analytic structural equation modelling, the association of job strain and depressive symptoms appears to be bi-directional, with both job strain predicting risk of depressive symptoms and vice versa. Given this, the observed association between job strain and hospital-treated depression might overestimate the causal effect of job strain on depression, although the association is unlikely to be fully attributable to confounding.

The precise pathways through which job strain may cause depression are unknown, but may involve social, behavioural and stress-physiological mechanisms. Previous studies have associated job strain with social isolation (Utzet *et al.* 2015), sleep disturbances (Linton *et al.* 2015) and leisure time physical inactivity (Fransson *et al.* 2012*b*; Griep *et al.* 2015) – all of which are known to be associated with increased risk of depression and somatic illnesses that may lead to depression (Barnett *et al.* 2007; Baglioni *et al.* 2011; Cooney *et al.* 2013). Some studies also suggest that exposure to chronic stressors, such as job strain, can cause dysregulation of the hypothalamic–pituitary–adrenal axis and subsequent physiological changes that are involved in the pathophysiology of depression, including loss of neuroplasticity, inhibition of neurogenesis, increased inflammation and disturbance of circadian rhythm (McEwen, 2004, 2012; Pittenger & Duman, 2007; Pariante & Lightman, 2008; Kronfeld-Schor & Einat, 2012; Gold, 2015). However, these hypotheses have not been examined in large-scale longitudinal studies.

### Strengths and limitations

The strengths of this study include the comprehensive approach of identifying all published data on job strain and depression and using a large individual participant dataset with assessment of job strain at the level of the individual, an objective outcome measure based on clinical diagnosis, and the pre-publication of a detailed study protocol pre-specifying the analyses. The large dataset provided sufficient power for examinations of effect modification. The register-based outcome data provided measurements based on clinical diagnoses and avoided common method bias (when both exposure and outcome are measured by self-reports), a potential bias in much previous research on job strain and depressive symptoms (Bonde, 2008; Netterstrøm *et al.* 2008; Siegrist, 2008; Theorell *et al.* 2015). The pre-published study protocol ensured that the analyses were not affected by *post-hoc* decisions, such as selective reporting, thus strengthening the validity of the findings.

There are some limitations to this study. All included studies measured job strain by self-report. Although this is the standard way to assess this exposure it is a potential limitation as the measurement may be influenced by the participants’ affective state. If participants’ affective state influenced both reporting of working conditions and subsequent risk of depression, this would cause reporting bias and inflated estimates (Kivimäki *et al.* 2010; Kolstad *et al.* 2011). All included studies were conducted in Europe or Canada, and the IPD-Work studies were further limited, with one exception, to the Nordic countries. We found no evidence for effect modification by country although given the small numbers of studies these tests are not powered to detect small or moderate differences between countries. Further research is needed to examine whether the present findings are generalizable beyond high-income or the Nordic countries. Our study focused on a specific aspect of the psychosocial work environment, job strain, which is the combination of high demands and low job control. We did not examine other psychosocial work stressors, such as effort–reward imbalance (Siegrist, 2016), the job demands–resources model (Bakker & Demerouti, 2016), job insecurity (Kim & von dem Knesebeck, 2015), job instability (Libby *et al.* 2010) or bullying at work (Verkuil *et al.* 2015). Moreover, it is possible that employee coping capacities modify the association between job strain and risk of depression. Further research is needed to examine whether adding these factors to the analyses of job strain would improve prediction of depression.

### Conclusions and clinical implications

We found consistent observational evidence that perceived job strain is associated with an increased risk of clinical depression. These data extend previous evidence that has largely been based on self-reported depressive symptoms.

Our findings have several clinical and research implications. First, clinicians should be aware that patients reporting job strain may be at an increased risk of depression and initiate relevant preventive measures or commence treatment as appropriate. The costs and benefits of following this recommendation need to be evaluated in future studies (Pignone *et al.* 2002; Palmer & Coyne, 2003; O'Connor *et al.* 2009). Second, further research is needed to determine if job strain represents a modifiable risk factor or only a risk marker for clinical depression. Given that the incidence of clinical depression is low in working populations, sufficiently powered randomized controlled trials on job strain and clinical depression would be costly and even unfeasible. However, trials to determine the potential of reducing job strain as a preventive measure for more common depression-related conditions, such as depressive symptoms, would be fruitful. Third, the identification and management of stress at work has become a legal imperative in many countries as set out in European Framework Directive 89/391/EEC (https://osha.europa.eu/en/legislation/directives/the-osh-framework-directive/1). Macro-level ecological studies, applying natural experiment designs to determine whether such policy measures are paralleled with favourable changes in depression incidence, would add to the evidence base regarding the potentially achievable reduction in depression by targeting job strain.

## Supplementary material

For supplementary material accompanying this paper visit http://dx.doi.org/10.1017/S003329171600355X. click here to view supplementary material
