# Supplementary material for: Job strain as a risk factor for clinical depression: systematic review and meta-analysis with additional individual participant data
Source: Psychol Med. 2017 Jan 26;47(8):1342–56. doi: 10.1017/S003329171600355X (PMC5471831; doi:10.1017/S003329171600355X)
Supplement: Supplementary file 1 [file S003329171600355Xsup001.doc]

**Supplementary material**

**Contents**

Supplementary Appendix S1: Inclusion of unpublished studies 2

Supplementary Appendix S2: Cohort descriptions for unpublished individual participant datasets 3

Supplementary Appendix S3: Ascertainment of hospital-treated depression in unpublished individual participant data 6

Supplementary Appendix S4: Overview of measures for baseline depressive symptoms in unpublished data 7

Supplementary Appendix S5: Flow chart for systematic review and characteristics of published studies 8

Supplementary Appendix S6: Quality assessment of included published studies 10

Supplementary Appendix S7: Sensitivity analysis with alternative job strain operationalizations in unpublished data 12

Supplementary Appendix S8: Repeat assessment of job strain in unpublished individual participant data 13

Supplementary Appendix S9: Temporal order of the association between job strain and depressive symptoms in unpublished individual participant data 14

Supplementary References 15

**Supplementary Appendix S1: Inclusion of unpublished studies**

The IPD-work consortium was established in 2008 at a meeting of principal investigators and researchers working on European cohort studies [1]. The consortium was established to advance research on work-related psychosocial factors and health. The overarching aim was to investigate the effect of work-related stress on chronic diseases using individual-participant data from prospective studies with a measure of work-related stress at baseline and register-based information on incident chronic diseases during follow-up.

Principal investigators of the studies were contacted in the 2008 meeting and additional cohorts that these investigators were aware of, we approached by meeting the principal investigators face-to-face or contacting them by telephone or email. With one exception (the HAPIEE study; the researchers did not have time and resources to participate), all studies originally contacted provided all the relevant individual-level data or conducted study-specific analyses according to our instructions and provided us with aggregate results. No study team refused to provide data or aggregate results.

Originally, 17 independent cohort studies were included in the IPD-work Consortium [1]. Of these 17 studies, 11 studies had relevant data for the current study and were included. The six studies not included were omitted because no register data on psychiatric hospital treatments were available (Belstress, GAZEL, Heinz-Nixdorf Recall study, Cooperative Health Research in the Region Augsburg (KORA) study, the Netherlands Working Conditions Survey (NWCS) and the Permanent Onderzoek Leefsituatie/ Continuous Survey on Living Conditions (POLS)). Furthermore, to maximize the number of participants in the analysis we included data from three subsequent waves of original IPD-work studies, where additional independent random samples were added, that is the Danish Work Environment Cohort Study (DWECS 2005), the Copenhagen Psychosocial Questionnaire study (COPSOQ-II) and the Swedish Longitudinal Occupational Survey of Health (SLOSH 2008), yielding a total of 14 included cohorts.

The selection of studies for the present analysis, based on the availability of register data on psychiatric hospital treatment may affect the generalizability of our results as the included studies were almost exclusively from Nordic countries. Besides this limitation, the selection of studies is unlikely to have biased the results of our meta-analysis.

A description of the included studies is given in Supplementary Appendix S2.

**Supplementary Appendix S2: Cohort descriptions for unpublished individual participant datasets**

**Copenhagen Psychosocial Questionnaire (COPSOQ)**

The COPSOQ-I is a prospective cohort study of a random sample of Danish residents selected from the Danish population register [2]. The participants were 20-60 years of age and were in paid employment at the study baseline in 1997. A baseline questionnaire and an invitation to take part was posted to 4 000 people and 2 454 individuals agreed to participate. There were 1 853 gainfully employed participants who were successfully linked with register data. We excluded 15 individuals with previous hospital treated depression and 123 due to missing data on job strain, sex, age, cohabitation or socioeconomic position, yielding a final sample of 1 715 participants.

COPSOQ-II [3] was carried out in 2004-2005. It included a follow up of respondents from COPSOQ I and a representative sample of Danish residents aged 20-60 at study baseline. The questionnaire was sent to 8 000 individuals and 4 732 individuals responded. The questionnaire could be completed via post or via the internet. There were 3 817 gainfully employed first time participants who were successfully linked with register data. We excluded 310 self-employed participants as job strain data were not collected for this group, a further 35 individuals with previous hospital treated depression, and 127 due to missing data on job strain, sex, age, cohabitation or socioeconomic position, yielding a final sample of 3 345 participants.

In Denmark, questionnaire- and register-based studies do not require approval from the Danish National Committee on Biomedical Research Ethics (Den Centrale Videnskabetiske komité). COPSOQ-I and COPSOQ II were approved by and registered with the Danish Data protection agency (registration numbers: 2008 - 54 -15 0553, 2004-54-1493).

**Danish Work Environment Cohort Study (DWECS)**

DWECS is a split panel survey of working age Danish people. [4,5] The cohort was established in 1990, when a simple random sample of men and women, aged 18-59, was drawn from the Danish population register. The participants have been followed up at five year intervals and data from the years 2000 and 2005 were used for the present analysis.

In 2000, 11 437 individuals were invited to participate and 8 583 agreed to do so. There were 5 606 individuals who were employed at baseline and were successfully linked with register data. We excluded 32 individuals with previous hospital treated depression and 123 due to missing data on job strain, sex, age, cohabitation or socioeconomic position, yielding a final sample of 5 451 participants. In 2005, an additional random sample of 8 545 individuals were invited to participate of which 5176 agreed to do so. There were 4 711 gainfully employed first-time participants in DWECS 2005 which were successfully linked with register data. We excluded 44 individuals with previous hospital treated depression and 230 due to missing data on job strain, sex, age, cohabitation or socioeconomic position, yielding a final sample of 4 437 participants. In Denmark, questionnaire- and register-based studies do not require ethics committee approval. DWECS was approved by and registered with the Danish Data protection agency (registration number: 2007-54-0059).

**Finnish Public Sector study (FPS)**

The Finnish Public Sector study is a prospective cohort study comprising the entire public sector personnel of 10 towns (municipalities) and 21 hospitals in the same geographical areas [6]. Participants, who were recruited from employers' records in 2000-2002, were individuals who had been employed in the study organizations for at least six months prior to data collection.^5^. There were 48 002 individuals aged 17 to 65 who responded to the questionnaire and were successfully linked with register data. We excluded 277 individuals with previous hospital treated depression and 1 530 due to missing data on job strain, sex, age, cohabitation or socioeconomic position, yielding a final sample of 46 195 participants. Ethical approval was obtained from the ethics committee of the Finnish Institute of Occupational Health.

**Health and Social Support (HeSSup)**

The Health and Social Support (HeSSup) study is a prospective cohort study of a stratified random sample of the Finnish population in the following four age groups: 20–24, 30–34, 40–44, and 50–54 [7]. The participants were identified from the Finnish population register and posted an invitation to participate, along with a baseline questionnaire, in 1998. Job strain was measured in 1998 and of the 25 898 individuals who responded to the questionnaire, 16 765 were in employment. We excluded 13 individuals with previous hospital treated depression and 1 580 due to missing data on job strain, sex, age, cohabitation or socioeconomic position, yielding a final sample of 15 172 participants. The Turku University Central Hospital Ethics Committee approved the study.

**Intervention Project on Absence and Well-being (IPAW)**

IPAW is a 5-year psychosocial work environment intervention study including 22 intervention and 30 control work places in three organizations (a large pharmaceutical company, municipal technical services and municipal nursing homes) in Copenhagen, Denmark [8]. The baseline questionnaire was posted to all the employees at the selected work sites between 1996 and 1997. Of the 2 721 employees who worked at the 52 IPAW sites, 2 055 men and women completed the baseline questionnaire and were successfully linked with register data. We excluded 21 individuals with previous hospital treated depression and 41 due to missing data on job strain, sex, age, cohabitation or socioeconomic position, yielding a final sample of 1 993 participants. IPAW was approved by and registered with the Danish Data Protection Agency (registration number: 2000-54-0066).

**Burnout, Motivation and Job Satisfaction study (Danish acronym: PUMA)**

Burnout, Motivation and Job Satisfaction study (Danish acronym: PUMA) is an intervention study of burn-out among employees in the human service sector [9]. Selection criteria for the participating organizations was that they had between 200 and 500 employees, that occupational groups within each organization were willing to participate and that the organizations would commit to the entire five-year study period. Participants gave consent to having their national identity numbers collected and used in later record linkages to Danish hospitalization and cause of death registries (Hospitalsindlæggelsesregisteret, Dødsårsagsregisteret). At study baseline in 1999-2000, 1 914 participants agreed to take part, of whom 1 905 were successfully linked with the registers. We excluded 19 individuals with previous hospital treated depression and 29 due to missing data on job strain, sex, age, cohabitation or socioeconomic position, yielding a final sample of 1 857 participants. PUMA was approved by the Scientific Ethical Committees (Videnskabsetisk Komiteer) in the counties in which the study was conducted and approved by and registered with the Danish Data Protection Agency (registration number: 2000-54-0048).

**Swedish Longitudinal Occupational Survey of Health (SLOSH)**

Swedish Longitudinal Occupational Survey of Health (SLOSH) is an on-going prospective cohort study following up individuals who participated in the Swedish Work Environment Survey (SWES) in 2003 or 2005 [10,11]. SWES, conducted biennially by Statistics Sweden, is based on a sample of gainfully employed people aged 16-64 years drawn from the Labour Force Survey (LFS). These individuals were first sampled into LFS through stratification by county, sex, citizenship and inferred employment status.

Data from the 2006 and 2008 data collection waves of SLOSH were used in the IPD-Work analyses. In both years, data were collected using postal self-completion questionnaires. In 2006, 5 985 individuals responded to the questionnaire. Of these, 5 149 people worked at least 30% of full time working hours. We excluded 58 individuals with previous hospital treated depression and 127 due to missing data on job strain, sex, age, cohabitation or socioeconomic position, yielding a final sample of 4 964 participants. In 2008, a further 6 751 individuals responded to the questionnaire. Of these, 5 904 men and women worked at least 30% of full time working hours. We excluded 90 individuals with previous hospital treated depression and 246 due to missing data on job strain, sex, age, cohabitation or socioeconomic position, yielding a final sample of 5 568 participants. SLOSH has been approved by the Regional Research Ethics Board in Stockholm.

**Still Working**

Still Working is an ongoing prospective cohort study. In 1986, the employees (n = 12 173) at all Finnish centers of operation of Enso Gutzeit (a forestry products manufacturer) were invited to participate in a questionnaire survey on demographic, psychosocial and health-related factors [12]. At baseline, 9 332 individuals responded. We excluded 50 individuals with previous hospital treated depression and 165 due to missing data on job strain, sex, age, cohabitation or socioeconomic position, yielding a final sample of 9 117 participants. The study was approved by the ethics committee of the Finnish Institute of Occupational Health.

**Whitehall II**

The Whitehall II study is a prospective cohort study set up to investigate socioeconomic determinants of health. At study baseline in 1985-1988, 10 308 civil service employees (6 895 men and 3 413 women) aged 35-55 and working in 20 civil service departments in London were invited to participate in the study [13]. We excluded 37 individuals due to missing data on job strain, sex, age, cohabitation or socioeconomic position, yielding a final sample of 10 271 participants. In Whitehall II register data on hospital treatment before baseline was unavailable and no prevalent cases were excluded. The Whitehall II study protocol was approved by the University College London Medical School committee on the ethics of human research. Written informed consent was obtained at each data collection wave.

**WOLF (Work, Lipids, and Fibrinogen) Stockholm and WOLF Norrland studies**

The WOLF (Work, Lipids, and Fibrinogen) Stockholm study is a prospective cohort study of 5 698 people (3 239 men and 2 459 women) [14,15]. We excluded 27 individuals with previous hospital treated depression and 187 due to missing data on job strain, sex, age, cohabitation or socioeconomic position, yielding a final sample of 5 484 participants. WOLF Norrland is a prospective cohort of 4 718 participants aged 19-65 working in companies in Jämtland and Västernorrland counties. We excluded 29 individuals with previous hospital treated depression and 47 due to missing data on job strain, sex, age, cohabitation or socioeconomic position, yielding a final sample of 4 642 participants. At study baseline the participants underwent a clinical examination and completed a set of health questionnaires. For WOLF Stockholm, the baseline assessment was undertaken at 20 occupational health units between November 1992 and June 1995 and for WOLF Norrland at 13 occupational health service units in 1996-98. The Regional Research Ethics Board in Stockholm, and the ethics committee at Karolinska Institutet, Stockholm, Sweden approved the study.

**Supplementary Appendix S3: Ascertainment of hospital-treated depression in unpublished individual participant datasets**

The register data on hospital treatment were included from national registers [16-19]. Danish register data were included from 1969 to 2012 and contained outpatient data from 1995 [16]. In Denmark, data were coded according to ICD-8 until 1994. ICD-9 was never implemented in Denmark, but ICD-10 was used from 1994 onwards [16]. Swedish register data for SLOSH were included from 1964 to 2011 and for WOLF from 1969 to 2008 and for SLOSH it contained outpatient data from 2001 [17]. In Sweden, ICD-8 was applied until 1987 when ICD-9 was introduced. ICD-10 was introduced in 1997, with the exception of the county of Skåne where ICD-9 was still in use throughout 1997 [17]. Finnish register data were included from 1980 to 2011 for FPS, from 1998 to 2005 for HeSSup, from 1972 to 2008 for Still Working and included only inpatient treatment. In Finland, diagnoses were recorded using the ICD-8 from 1969 to 1986, ICD-9 from 1987 to 1995, and ICD-10 from 1996 onwards [18]. UK register data were included from 1989 to 2012. Only inpatient treatment data were used in the current study. The coverage of these data before 2004 is low and given the year of study baseline (1985-1988) it was not possible to exclude participants with previous depression in the Whitehall II study. UK data were coded according to ICD-9 until 1994-95 and using ICD-10 from 1995-96 onwards [19].

**Supplementary Table S1. *Definition of depression***

| **Classification** | **Codes** | **Name of codes** |
| --- | --- | --- |
| **ICD-10 (primary definition)** | F32; F33 | Depressive episode; Recurrent depression |
| **ICD-9 (mainly used to exclude participants with depression before baseline)** | 296.2; 296.3;  298.0; 311 | Major depressive disorder, single episode; Major depressive disorder, recurrent episode; Depressive type psychosis; Depressive disorder not elsewhere classified. |
| **ICD-8 (only used to exclude participants with depressive disorder before baseline)** | 296.0; 296.2;  298.0, 300.4 | Involutional melancholia; Manic-depressive psychosis, depressed; Reactive depressive psychosis; Depressive neurosis. |
| For Finland and Sweden, we took into account the national coding in the ICD-9 definition and included: 296.1/ 296B Unipolar affective psychosis, melancholia; 298.0 / 298A Depressive type psychosis; 300.4/ 300E Neurotic depression; 311 Depressive disorder not elsewhere classified. ICD-9 was never applied in Danish hospital registers. | | |

**Supplementary Appendix S4: Overview of measures for baseline depressive symptoms in unpublished data**

***Supplementary Table S2.*** Measures for baseline depressive symptoms

| **Study^a^** | **Measure of depressive symptoms at baseline** | **Source** |
| --- | --- | --- |
| **COPSOQ I** | Mental health inventory (MHI-5) | The Short Form Health Survey [20] |
| **COPSOQ II** | Depressive symptoms | The Copenhagen Psychosocial Questionnaire II [3] |
| **DWECS 2000 and DWECS 2005** | Mental health inventory (MHI-5) | The Short Form Health Survey [20] |
| **FPS** | General Health Questionnaire (GHQ-12) | General Health Questionnaire [21] |
| **HeSSup** | Depressive symptoms | Beck depression inventory [22] |
| **IPAW** | Mental health inventory (MHI-5) | The Short Form Health Survey [20] |
| **PUMA** | Mental health inventory (MHI-5) | The Short Form Health Survey [20] |
| **SLOSH 2006** | Depressive symptoms | Symptom Check List, 6 item subscale [23,24] |
| **SLOSH 2008** | Depressive symptoms | Symptom Check List, 6 item subscale [23,24] |
| **Whitehall II^b^** | General Health Questionnaire (GHQ-12) | General Health Questionnaire^16^ |
| ^a^Study acronyms: COPSOQ: Copenhagen Psychosocial Questionnaire Study, DWECS: Danish Work Environment Cohort Study, FPS: Finnish Public Sector study, HeSSUP: Health and Social support Study, IPAW: Intervention Project on Absence and Well-being, PUMA: Burnout, Motivation and Job Satisfaction study, SLOSH: Swedish Longitudinal Occupational Survey of Health.  ^b^We applied the full GHQ-12 scale in adjusting for baseline depressive symptoms in the Whitehall II study, instead of the depressive symptoms subscale that was outlined in the protocol. This decision was based on the content of the depressive symptoms subscale, which focused mainly on suicidal ideation, and thus measured more severe symptoms than the other included scales. | | |

**Supplementary Appendix S5: Flow chart for systematic review**

**Supplementary Fig. S1.** Flow chart for systematic review.

**Screening**

**Included**

**Eligibility**

**Identification**

852 Records identified through database searching

418 PubMed

434 PsycNET

283 Additional records identified through other sources
 274 Web of Science Citations

9 Hand search

988 Records after duplicates removed

988 Records screened

956 Records excluded

32 Full-text articles assessed for eligibility

26 Full-text articles excluded

6 Duplicate cohorts

10 Cross-sectional

5 Not individual level job strain

5 Not clinical depression

6 Studies included in meta-analysis

6 Studies eligible for inclusion
3 studies where published data sufficient for meta-analysis

3 studies where investigators provided relevant risk estimates

**Supplementary Appendix S6: Quality assessment of included published studies**

**NEWCASTLE - OTTAWA QUALITY ASSESSMENT SCALE**

**COHORT STUDIES**

Note: A study can be awarded a maximum of one star for each numbered item within the Selection and Outcome categories. A maximum of two stars can be given for Comparability

**Selection**

1) Representativeness of the exposed cohort

a) truly representative of the average working population in the country **🟑**

b) somewhat representative of the average working population in the country **🟑**

c) selected group of users e.g. nurses, volunteers

d) no description of the derivation of the cohort

2) Selection of the non exposed cohort

a) drawn from the same community as the exposed cohort **🟑**

b) drawn from a different source

c) no description of the derivation of the non exposed cohort

3) Ascertainment of exposure

a) secure record (e.g. surgical records) **🟑**

b) structured interview **🟑**

c) written self report

d) no description

4) Demonstration that outcome of interest was not present at start of study

a) yes **🟑**

b) no

**Comparability**

1) Comparability of cohorts on the basis of the design or analysis

a) study controls for sex (select the most important factor) **🟑**

b) study controls for age **🟑** (This criteria could be modified to indicate specific control for a second important factor.)

**Outcome**

1) Assessment of outcome

a) independent blind assessment **🟑**

b) record linkage **🟑**

c) self report

d) no description

2) Was follow-up long enough for outcomes to occur

a) yes, at least 12 months between baseline and follow up**🟑**

b) no

3) Adequacy of follow up of cohorts

a) complete follow up - all subjects accounted for **🟑**

b) subjects lost to follow up unlikely to introduce bias - small number lost - > 80 % follow up, or description provided of those lost) **🟑**

c) follow up rate < 80% (select an adequate %) and no description of those lost

d) no statement

***Supplementary Table S3.*** *Quality assessment of included published studies*

| **Study** | **Selection** |  |  |  | **Comparability** | **Outcome** |  |  | **Total rating** | **Quality assessment^a^** |
| --- | --- | --- | --- | --- | --- | --- | --- | --- | --- | --- |
|  | Representativeness of the exposed cohort | Selection of the non exposed cohort | Ascertainment of exposure | Demonstration that outcome of interest was not present at start of study | Comparability of cohorts on the basis of the design or analysis | Assessment of outcome | Was follow up long enough for outcome to occur | Adequacy of Follow-up of cohorts |  |  |
| Grynderup,2012^25^ | C | A * | C | A * | A, B ** | A * | A* | C | 6 * | Fair |
| Niedhammer, 2015^26^ | B * | A * | C | A * | A, B ** | A * | A * | B * | 8 * | Good |
| Shields, 2006^27^ | B * | A * | C | A * | A, B ** | A * | A * | B * | 8 * | Good |
| Virtanen, 2012^28^ | C | A * | C | B | A, B ** | A * | A * | B * | 6 * | Poor |
| Wang, 2012^29^ | B * | A * | C | A * |  | A * | A * | C | 5 * | Poor |
| Plaisier, 2007^30^ | B * | A * | C | A * | A, B ** | A * | A * | B * | 8* | Good |
| ^a^Categorized as:  Good quality: 3 or 4 stars in selection domain AND 1 or 2 stars in comparability domain AND 2 or 3 stars in outcome/exposure domain  Fair quality: 2 stars in selection domain AND 1 or 2 stars in comparability domain AND 2 or 3 stars in outcome/exposure domain  Poor quality: 0 or 1 star in selection domain OR 0 stars in comparability domain OR 0 or 1 stars in outcome/exposure domain | | | | | | | | | | |

**Supplementary Appendix S7: Sensitivity analysis with alternative job strain operationalizations in unpublished data**

In this analysis we used quadrants of job strain and continuous scores of job demands and job control instead of the binary variable job strain versus no job strain.

***Supplementary Table S4.*** *Association of alternative definitions of job strain with* hospital treated depression, adjusted for age, sex and cohabitation

|  |  | **Hazard Ratio** | **95% CI** | |
| --- | --- | --- | --- | --- |
| **Quadrants of job strain** | Low strain | 1.00 | reference | |
|  | Job strain | 1.35 | 1.08-1.70 |  |
|  | Passive | 1.23 | 1.04-1.45 |  |
|  | Active | 1.02 | 0.85-1.23 |  |
|  |  |  |  |  |
| **Continuous demands and control** | Demands, per SD increase | 1.03 | 0.96-1.12 |  |
|  | Control, per SD increase | 0.87 | 0.79-0.95 |  |
|  | Demands*Control, per SD increase | 0.96 | 0.91-1.02 |  |

**Supplementary Appendix S8: Repeat assessment of job strain in unpublished individual participant data**

This analysis uses two (repeated) measurements of job strain, on average 4.8 years apart, and follow up for hospital treated depression starts after the second measurement. Data used are from DWECS, FPS, HeSSup, IPAW, PUMA, Still working, and Whitehall II for the number of job strain reports (COPSOQ, SLOSH, and WOLF-N were excluded due to lack of cases in some exposure categories) and FPS, HeSSup, IPAW, PUMA, Still working for the changes in job strain (DWECS and Whitehall II also excluded due to lack of cases in some exposure categories)

***Supplementary Table S5.*** *Age, sex and cohabitation adjusted association between repeatedly assessed* job strain and hospital treated depression

| **Number of job strain reports** | **N (total)** | **N (cases)** | **Hazard ratio** | **95% CI** |
| --- | --- | --- | --- | --- |
| **0** | 39732 | 152 | 1.00 | reference |
| **1** | 9807 | 47 | 1.23 | 0.88 - 1.71 |
| **2** | 3470 | 21 | 1.56 | 0.99- 2.45 |
| **Test for trend*** |  |  | *P* = 0.033 |  |
|  |  |  |  |  |
| **Changes in job strain** |  |  |  |  |
| **No strain T_1_ - No strain T_2_** | 31911 | 125 | 1.00 | reference |
| **Strain T_1_ - No strain T_2_** | 3898 | 16 | 1.12 | 0.66-1.89 |
| **No strain T_1_ - Strain T_2_** | 3788 | 21 | 1.22 | 0.77-1.94 |
| **Strain T_1_ - Strain T_2_** | 2870 | 18 | 1.63 | 0.99-2.68 |
|  |  |  |  | |

*Hazard ratio per additional report of job strain 1.24 (95% CI: 1.02-1.52)

**Supplementary Appendix S9: Temporal order of the association between job strain and depressive symptoms in unpublished individual participant data**

Attenuation of the job strain-hospital treated depression association after adjustment for depressive symptoms at baseline may be explained by mediation or confounding by depressive symptoms. The mediation hypothesis is that job strain increases the risk of depressive symptoms and eventually hospital-treated depression. In this scenario, adjusting the association between job strain and hospital treated depression for depressive symptoms represents over-adjustment. The confounding hypothesis, in turn, is that depressive symptoms affect perception of job strain and increase risk of hospital-treated depression. In this scenario, adjustment for depressive symptoms is indicated. We examined these alternative hypotheses by testing temporal order between job strain and depressive symptoms. Results presented in Supplementary Table S6 suggest bi-directional associations between job strain and depressive symptoms: Job strain in participants free of depressive symptoms at baseline is associated with an increased risk of depressive symptoms at follow-up, supporting the mediation hypothesis. However, in participants without job strain at baseline, depressive symptoms are associated with an increased risk of job strain at follow-up, consistently with the confounding hypothesis.

***Supplementary Table S6.*** *Age, sex and cohabitation adjusted associations between baseline job strain and depressive symptoms at follow-up and between depressive symptoms at baseline and job strain at follow-up*

| **Subpopulation and exposure** | **Outcome at follow-up** | **N participants (N cases)** | **Relative risk** | **95% CI** |
| --- | --- | --- | --- | --- |
| Participants free of depressive symptoms at baseline | New onset depressive symptoms |  |  |  |
| No job stain |  | 43771 (5486) | 1.00 | Reference |
| Job strain |  | 7394 (1341) | 1.39 | 1.23- 1.57 |
| Participants without job strain at baseline | New onset job strain |  |  |  |
| No depressive symptoms |  | 42946 (4147) | 1.00 | Reference |
| Depressive symptoms |  | 7787 (1146) | 1.46 | 1.36-1.57 |

Note. Analyses are based on DWECS, FPS, HeSSuP, IPAW, PUMA, SLOSH 2006, SLOSH 2008,Whitehall II.

Bi-directional associations between job strain and depressive symptoms were also supported by the meta-analytic structural equation modeling as shown in Supplementary Fig. S2, with a similar strength of the association between job strain at baseline and depressive symptoms at follow up, as the reverse association between depressive symptoms at baseline and job strain at follow up. The model is based on data from DWECS, FPS, HeSSuP, IPAW, PUMA, SLOSH 2006, SLOSH 2008,Whitehall II (n=58,234), and showed good fit (RMSEA=0. 0.0282, 95% CI: 0.0248-0.0317; SRMR = 0.0145).

**
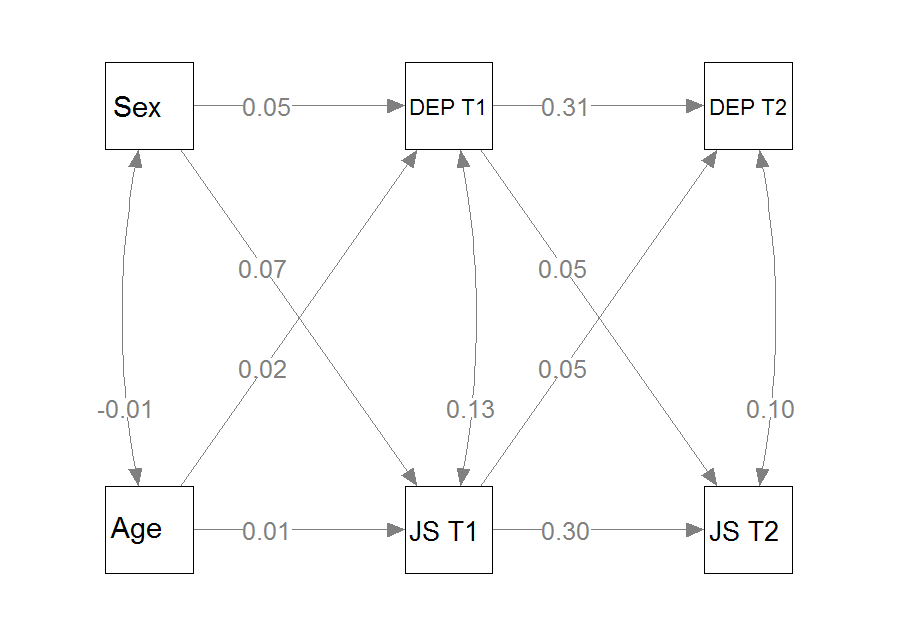
**

**Supplementary Fig. S2.** Meta-analytic structural equation modeling of cross-lagged associations between job strain (JS) and depressive symptoms (DEP).

**Supplementary References**

1. Kivimäki M, Singh-Manoux A, Virtanen M, Ferrie JE, Batty GD, Rugulies R. IPD-Work consortium: pre-defined meta-analyses of individual-participant data strengthen evidence base for a link between psychosocial factors and health. Scand J Work Environ Health. 2015, Feb 5 (online first).
2. Kristensen TS, Hannerz H, Høgh A, Borg V. The Copenhagen psychosocial questionnaire--a tool for the assessment and improvement of the psychosocial work environment. Scand J Work Environ Health 2005;31(6):438-49.
3. Pejtersen JH, Kristensen TS, Borg V, Bjorner JB. The second version of the Copenhagen Psychosocial Questionnaire. Scand J Public Health 2010;38:8-24.
4. Burr H, Bjorner JB, Kristensen TS, Tüchsen F, Bach E. Trends in the Danish work environment in 1990-2000 and their associations with labor-force changes. Scand J Work Environ Health 2003;29(4):270-9.
5. Feveile H, Olsen O, Burr H, Bach E. Danish work environment cohort study 2005: from idea to sampling design. Statistics in Transition 2007;8(3):441-58.
6. Kivimäki M, Lawlor DA, Smith GD, Kouvonen A, Virtanen M, Elovainio M et al. Socioeconomic position, co-occurrence of behavior-related risk factors, and coronary heart disease: the Finnish public sector study. Am J Public Health 2007;97(5):874-9.
7. Korkeila K, Suominen S, Ahvenainen J, Ojanlatva A, Rautava P, Helenius H et al. Non-response and related factors in a nation-wide health survey. Eur J Epidemiol 2001;17(11):991-9.
8. Nielsen ML, Kristensen TS, Smith-Hansen L. The Intervention Project on Absence and Well-being (IPAW): Design and results from the baseline of a 5-year study. Work and Stress 2002;16(3):191-206.
9. Borritz M, Rugulies R, Bjorner JB, Villadsen E, Mikkelsen OA, Kristensen TS. Burnout among employees in human service work: design and baseline findings of the PUMA study. Scand J Public Health 2006;34(1):49-58.
10. Magnusson Hanson L, Theorell T, Oxenstierna G, Hyde M, Westerlund H. Demand, control and social climate as predictors of emotional exhaustion symptoms in working Swedish men and women. Scand J Public Health 2008;36(7):737-43.
11. Leineweber L, Baltzer M, Magnusson Hanson L, Westerlund H. Work–family conflict and health in Swedish working women and men: a 2-year prospective analysis (the SLOSH study). Eur J Public Health 2013; 23(4):710-16.
12. Väänänen A, Murray M, Koskinen A, Vahtera J, Kouvonen A, Kivimäki M. Engagement in cultural activities and cause-specific mortality: Prospective cohort study. Preventive Medicine 2009;49(2-3):142-7.
13. Marmot M, Brunner E. Cohort profile: The Whitehall II study. Int J Epidemiol 2005;34(2):251-6.
14. Alfredsson L, Hammar N, Fransson E, de Faire U, Hallqvist J, Knutsson A et al. Job strain and major risk factors for coronary heart disease among employed males and females in a Swedish study on work, lipids and fibrinogen. Scand J Work Environ Health 2002:238-48.
15. Peter R, Alfredsson L, Hammar N, Siegrist J, Theorell T, Westerholm P. High effort, low reward, and cardiovascular risk factors in employed Swedish men and women: Baseline results from the WOLF study. J Epidemiol Community Health 1998;52(9):540-7.
16. Mors O, Perto GP, Mortensen PB. The Danish Psychiatric Central Research Register. Scand J Public Health 2011;39: 54-57.
17. Ludvigsson J, Andersson E, Ekbom A, Feychting M, Kim JL, Reuterwall C et al. External review and validation of the Swedish national inpatient register. BMC Public Health 2011;11(1):450.
18. Sund R. Quality of the Finnish Hospital Discharge Register: A systematic review. Scand J Public Health 2012 August 1;40(6):505-15
19. Health and Social Care Information Centre. Hospital Episode Statistics (HES) Analysis Guide. Available from: www.hscic.gov.uk/hesdata
20. Bjorner JB, Damsgaard MT, Watt T, Groenvold M. Tests of data quality, scaling assumptions, and reliability of the Danish SF-36. J Clin Epidemiol 1998;51(11):1001-11.
21. Goldberg DP, Hillier VF. A scaled version of the General Health Questionnaire. Psychol Med 1979;9(1):139-45.
22. Beck AT, Ward CH, Mendelson M, Mock J, Erbaugh J. An inventory for measuring depression. Arch Gen Psychiatry 1961;4:561-71.
23. Lipmann R. Depression scales derived from Hopkins Symptom Checklist. In: Sartorius N, Bann T, editors. Assessment of depression. Berlin: Springer; 1986. p. 232-48.
24. Magnusson Hanson L, Theorell T, Bech P, Rugulies R, Burr H, Hyde M et al. Psychosocial working conditions and depressive symptoms among Swedish employees. Int Arch Occup Environ Health 2009;82(8):951-60.
25. Grynderup MB, Mors O, Hansen AM et al. A two-year follow-up study of risk of depression according to work-unit measures of psychological demands and decision latitude. Scand J Work Environ Health. 2012;38(6):527-536.
26. Niedhammer I, Malard L, Chastang JF. Occupational factors and subsequent major depressive and generalized anxiety disorders in the prospective French national SIP study. BMC Public Health 2015;15(1):200.
27. Shields M. Stress and depression in the employed population. Health Rep. 2006;17(4):11-29.
28. Virtanen M, Stansfeld SA, Fuhrer R, Ferrie JE, Kivimäki M. Overtime Work as a Predictor of Major Depressive Episode: A 5-Year Follow-Up of the Whitehall II Study. Plos One. 2012;7(1).
29. Wang J, Patten SB, Currie S, Sareen J, Schmitz N. A population-based longitudinal study on work environmental factors and the risk of major depressive disorder. Am J Epidemiol. 2012;176(1):52-59.
30. Plaisier I, de Bruijn JGM, de Graaf R, ten Have M, Beekman ATF, Penninx BWJH. The contribution of working conditions and social support to the onset of depressive and anxiety disorders among male and female employees. Soc Sci Med. 2007;64(2):401-410.
